# **Value signals guiding choices for cannabis versus non-drug rewards in people who use cannabis near-daily**

**DOI:** 10.1007/s00213-025-06746-6

**Published:** 2025-02-10

**Authors:** Will Lawn, Xuejun Hao, Anna B. Konova, Margaret Haney, Ziva D. Cooper, Nicholas Van Dam, Paul Glimcher, Gillinder Bedi

**Affiliations:** 1 Department of Psychology, Institute of Psychiatry Psychology and Neuroscience, King’s College London, London, UK; 2 Clinical Psychopharmacology Unit, University College London, London, UK; 3 Department of Psychiatry, Columbia University Medical Center and New York State Psychiatric Institute, New York, NY USA; 4 Department of Psychiatry, Brain Health Institute, Rutgers University Behavioral Health Care, Rutgers University, Piscataway, NJ USA; 5 UCLA Center for Cannabis and Cannabinoids, Jane and Terry Semel Institute for Neuroscience and Human Behavior, Department of Psychiatry and Biobehavioral Sciences, David Geffen School of Medicine, University of California, Los Angeles, USA; 6 Contemplative Studies Centre, Melbourne School of Psychological Sciences, University of Melbourne, Melbourne, Australia; 7 Institute for the Interdisciplinary Study of Decision Making, New York University, New York, NY USA; 8 Centre for Youth Mental Health, University of Melbourne, and Orygen, Melbourne, Australia

**Keywords:** Cannabis, Decision-making, Choice, Subjective value, FMRI, Neuroeconomics

## Abstract

**Rationale:**

Despite the critical role of choice processes in substance use disorders, the neurobehavioral mechanisms guiding human decisions about drugs remain poorly understood.

**Objectives:**

We aimed to characterize the neural encoding of subjective value (SV) for cannabis versus non-drug rewards (snacks) in people who use cannabis on a near-daily/daily frequency (PWUCF) and assessed the impact of cannabis and snack stimuli (‘cues’) on SV encoding.

**Methods:**

Twenty-one non-treatment-seeking PWUCF (≥4 days/week; 1 female) participated in an inpatient, crossover experiment with four counterbalanced conditions: 1. neutral cues/cannabis choices; 2. cannabis cues/cannabis choices; 3. neutral cues/snack choices; and 4. snack cues/snack choices. In each condition, participants were exposed to cues before an fMRI scan during which they repeatedly chose between 0-6 cannabis puffs/snacks and a set monetary amount, with randomly-selected choices implemented. The SV signal was operationalized as the neural correlates of the strength of preference for cannabis/snack choices. fMRI data were analyzed for twenty participants.

**Results:**

Despite equivalent choice behavior, SV signals for cannabis, but not snacks, were observed in regions known to encode SV for various rewards (ventromedial prefrontal cortex, vmPFC; ventral striatum; dorsal posterior cingulate cortex, dPCC). SV encoding in vmPFC was stronger for cannabis than snacks. In the dPCC, the impact of cues on SV signals was moderated by reward type.

**Conclusions:**

PWUCF had expected neural value encoding for cannabis but disrupted non-drug SV encoding, despite equivalent choice behavior. This provides tentative support for theories that highlight dysregulated neural valuation of non-drug rewards as a hallmark of problematic cannabis use.

**Supplementary Information:**

The online version contains supplementary material available at 10.1007/s00213-025-06746-6.

## Introduction

Persistent choice for drugs at the expense of healthier rewards is a cardinal pathology in substance use disorders (SUDs) (American Psychiatric Association 2013). Indeed, it has been argued that SUDs are, in essence, pathologies of choice and decision-making (Bickel et al. 2011, 2014; Ekhtiari et al. 2017; Redish et al. 2008). A fundamental question about SUDs thus concerns the neurobehavioral mechanisms guiding these choices: why do decisions to use drugs continue, often despite negative consequences?

Novel approaches to this question have recently come from the fields of neuroeconomics and decision science, which have made substantial progress in characterizing the neural processes guiding choices for various rewards in healthy humans (Hare et al. 2008, 2011; Levy and Glimcher 2011, 2012). A major focus has been neural representation of *subjective value* (SV). This emerges from a fundamental computational problem that must be resolved by the brain during choices: how to compare qualitatively different options (Levy and Glimcher 2011). A central tenet of economics is that individuals make choices ‘as if’ using a single SV scale, calculated as a ‘common currency’. Moreover, economists hold that this SV scale is revealed in the choices themselves (Glimcher 2010), such that an individual who chooses a bag of cannabis over $10 must, by definition, have a greater SV for that amount of cannabis than for $10, under those precise circumstances.

Within the past 20 years neuroeconomics has investigated the neurobiological basis of SV calculations in healthy populations. Intriguingly, results indicate that the brain appears to calculate SV signals as predicted by standard economic theory (Bartra et al. 2013; Clithero and Rangel 2014). There is strong evidence that SV signals are parametrically encoded during decisions in the ventromedial Prefrontal Cortex (vmPFC) (Chib et al. 2009; Clithero and Rangel 2014; Glimcher 2010), the Ventral Striatum (VS), and dorsal Poster Cingulate Cortex (dPCC) (Bartra et al. 2013; Clithero and Rangel 2014). These findings are particularly relevant for addiction, given that individuals with SUDs are believed to overvalue drugs while simultaneously undervaluing other rewards (Ahmed et al. 2020; Goldstein and Volkow 2011; Hogarth and Field 2020; Verdejo-Garcia 2014). Dysregulated encoding of SV may thus be a key substrate for continued decisions to take drugs. A small number of studies have investigated the neural correlates of drug-related decisions (Bedi et al. 2015; Gray et al. 2017; Lawn et al. 2020; MacKillop et al. 2012, 2014). To our knowledge, only one has investigated neurobiological SV signals during drug-related decision-making, and that was in cigarette smokers (Lawn et al. 2020). SV encoding for other substances remains unexamined.

A second key outstanding question concerns the degree to which neural processing underlying drug-related decisions differs from that during choices about other rewards. Blunted valuation of non-drug rewards coupled with heightened drug valuation is theorized to contribute to the maintenance of SUDs (Goldstein and Volkow 2011). The only prior study assessing this question observed, in frequent cigarette smokers, normative SV signals for cigarettes but not for shop vouchers (Lawn et al. 2020). A preferential disruption to valuation processes underlying non-drug versus drug reinforcers may influence treatment response (Field et al. 2020; Lubman et al. 2009; Versace et al. 2012), especially for treatments like contingency management, which requires that patients be motivated by non-drug rewards (Petry et al. 2017).

A third question concerns the mechanisms underlying the influence of drug-related cues on choices for drugs. In preclinical models, exposure to drug-paired stimuli elicits drug-seeking (van den Oever et al. 2010). In humans, conditioned drug-related stimuli (‘cues’) elicit craving, and cue exposure usually, though not always, increases drug-taking (Haney 2009; Hogarth et al. 2010; Hogarth and Field 2020). Despite the link between cue exposure and drug use, no research studies investigating the impact of cues on SV encoding during drug choice have been conducted.

Here, we used a highly-controlled experimental medicine approach to address these questions in a population that uses cannabis frequently. We studied cannabis-related choices because cannabis is the most commonly used controlled recreational drug globally, and cannabis use disorder (CUD) continues to rise against a backdrop of changes to the drug’s legality (Cerdá et al. 2020; Chiu et al. 2021). In people who use cannabis frequently (PWUCF), we aimed to examine: (1) drug (cannabis) neural SV signals relative to (2) non-drug reward (snack food) neural SV signals, and (3) the influence of cues (cannabis, snack food, neutral) on drug and non-drug reward neural SV signals. We hypothesized that: (1) SV signals for both cannabis and snacks would be found in the vmPFC, VS, and dPCC; (2) SV signals would be stronger during choices about cannabis compared to snack food; and (3) the presence of cues would augment the cannabis SV signals more than snack SV signals.

## Materials and methods

### Participants

We recruited 21 right-handed 21–50-year-old participants (one female; 20 males) who smoked cannabis in ‘blunts’ or ‘joints’ ≥4 days/week. One participant’s fMRI data was lost, so we included 20 participants in fMRI analyses and 21 participants in behavioral analyses. None were seeking treatment for cannabis use, and none used other illicit drugs > 2 days/week. They had no DSM-IV Axis 1 disorder requiring intervention, were not seeking treatment for mental health or other conditions, and had no contraindications for participating. Full eligibility criteria and CONSORT diagram are in the Supplement (Table S9). Participants provided written informed consent and were compensated as approved by the New York State Psychiatric Institute’s (NYSPI) Institutional Review Board.

### Experimental protocol

Following screening and training in the tasks and procedures, participants were admitted for six days to the Clinical Research Unit at NYSPI (Table 1). Urine toxicology, carbon monoxide, and breathalyzer tests confirmed sobriety at arrival. Upon admission, participants underwent two sessions, wherein they sampled the cannabis (6 puffs) and personally-selected snacks (6 snacks) about which they would make decisions. Snack sessions always occurred before cannabis sessions.

**Table 1:** Representative Study schedule

Day	Admission	Day 1	Day 2	Days 3 & 4	Day 5	Day 6
Condition	Move in	Neutral cues & snack choices	Cannabis cues & cannabis choices	N/A	Neutral cues & cannabis choices	Snack cues & snack choices
Cues	N/A	Neutral	Cannabis	N/A	Neutral	Snacks
Behavioral Choice Task	N/A	Snacks vs. $$	Cannabis vs. $$	N/A	Cannabis vs. $$	Snacks vs. $$
fMRI Choice Task	N/A	Snacks vs. $$	Cannabis vs. $$	N/A	Cannabis vs. $$	Snacks vs. $$
Administration Session 1	Snack sampling	Snack or $$^3^	Cannabis or $$^3^	Cannabis	Cannabis or $$^3^	Snack or $$^3^
Administration session 2	Cannabis sampling	Snack or $$^4^	Cannabis or $$^4^	N/A	Cannabis or $$^4^	Snack or $$^4^
Cannabis doses^1^	6	0	Up to 12	6^5^	Up to 12	0
Snacks^2^	6	Up to 12	0	0	0	Up to 12
EA or SA	EA	SA	SA	EA	SA	SA

The order of conditions was counterbalanced across participants. EA = experimenter-administered (i.e., no choice required); SA = self-administered dependent on choices made. $$=money. ^1^1 dose = 1 puff of 5.6% THC cannabis; ^2^1 snack = 1 50–100 kcal. ^3^Administration session 1 on choice days involved administration of the chosen reward from one randomly selected decision made during the behavioral choice task. If in the selected item, the participant chose money, they received that amount of money and did not undergo the first administration session. ^4^Administration session 2 on choice days involved administration of the chosen reward from one randomly selected decision made during the fMRI choice task. If in the selected item, the participant chose money, they received that amount of money and did not undergo the second administration session. ^5^Participants were administered six puffs of cannabis on weekend (non-choice) days to prevent the onset of cannabis withdrawal. They were also maintained on a normal (i.e., palatable) diet on weekends, whereas on choice days they received unlimited amounts of low fat, salt, and sugar food to ensure the snacks were reinforcing

On inpatient days 1, 2, 5 and 6 (‘choice’ days), participants underwent four conditions in counterbalanced order, with one condition daily: (1) cannabis (i.e., active) cues, cannabis versus money choices; (2) neutral cues, cannabis versus money choices; (3) snack (i.e., active) cues, snack versus money choices; and (4) neutral cues, snack versus money choices. They were reminded that: (1) the cannabis or snacks would be identical to those sampled; (2) they would complete two tasks (behavioral and fMRI), making repeated choices between cannabis/snacks and money; (3) one choice *per task* (two in total – one from the behavioral and one from the fMRI task) would be randomly selected and implemented that afternoon; and (4) because any choice could be implemented, the best strategy was to treat each choice as real. This approach ensures that choices have meaningful consequences and are perceived as independent (Chib et al. 2009; Hare et al. 2009).

The schedule (Table 1) was timed to avoid cannabis withdrawal onset, ensuring that participants would find the cannabis reinforcing but that choices would not be impacted by withdrawal. On choice days, participants were maintained on a low salt, sugar and fat diet, i.e., they were not hungry, but the snacks would remain reinforcing. Participants knew that the only way to receive cannabis or palatable food on choice days was via their decisions.

Table S1 presents a schedule for choice days. Participants underwent visual cue exposure and then completed the behavioral choice task, which informed the individualized monetary alternative for the subsequent fMRI task. They were then escorted to the NYSPI MRI, where they were exposed to multisensory cues, before completing the fMRI choice task.

That afternoon, participants completed up to two cannabis/snack sessions, in which they received the rewards from the two randomly-selected decisions implemented. If their choice in an implemented decision was money, they received the money and had no cannabis/snack session.

### Rewards: Cannabis and snack Sessions

Cannabis (5.6% ∆9- tetrahydrocannabinol; <0.1% cannabidiol) was administered using standardized paced puffing from a cannabis cigarette (Foltin et al. 1987). Each cigarette contained approximately 800 mg of cannabis and thus 44.8 mg of THC (~ 9 standard THC units) (Freeman and Lorenzetti 2020). Up to six puffs were administered in each session; one every 14 min (Bedi et al. 2015; Haney et al. 2013). Each puff was approximately 7.5 mg THC (~ 1.5 standard THC units). During snack sessions, participants ate their selected snacks at 14-minute intervals. Each snack had 50–100 calories.

Subjective mood was measured repeatedly during cannabis/snack sessions (see Supplement).

### Cues

Visual cues preceding the behavioral task comprised 30 pictures of cannabis or people smoking cannabis (cannabis cues), snacks or people eating snacks (snack cues), or neutral images, e.g., furniture (neutral cues). Multisensory cues preceding the fMRI task comprised visual, olfactory, tactile, and auditory stimuli. For cannabis, participants rolled a blunt/joint and smelled it. For snacks, they unwrapped, smelled and touched the snacks. For neutral cues, participants touched and smelled a scented candle and then gift-wrapped it (see Supplement).

Subjective effects of cues were measured with Visual Analogue Scales (VAS) before and after both the visual and multisensory cues.

### Behavioral choice tasks

The behavioral choice tasks served to determine the fixed, individualized monetary alternative for that day’s fMRI choice task, which was calculated as the estimated indifference point for 3 cannabis puffs or 3 snacks i.e., the monetary value equivalent to the worth of 3 puffs/snacks for that participant on that day (see Supplement). Participants made 30 choices between 0 and 6 puffs/snacks (dependent on condition) and monetary values from $0 to $30 to enable this calculation.

### fMRI choice tasks

We used novel event-related fMRI tasks, based on previous work (Hare et al. 2009), in which participants repeatedly chose between 0 and 6 cannabis puffs/snacks (dependent on condition) and the fixed monetary alternative determined in the behavioral task. A stable monetary alternative was used so that fMRI signal changes could be attributed to the changing SV of the cannabis/snack options. The individualized monetary value for 3 puffs/snacks was used as the alternative to ensure that choices would vary, and to approximately normalize choice behavior in the fMRI tasks so that differences in SV encoding could not be attributed to differences in behavior.

**Fig. 1:**
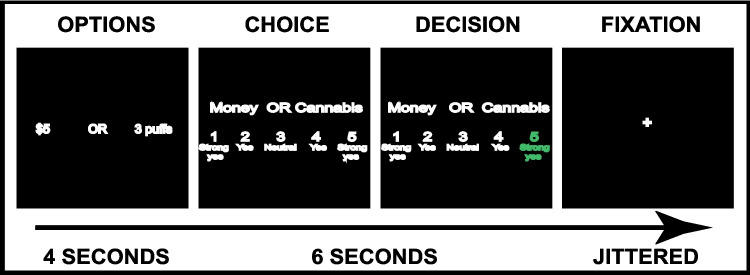
Schematic of fMRI Choice task. Each run consisted of 35 choices between 0–6 puffs/snacks and a fixed monetary alternative (set to the indifference point for 3 puffs/3 snacks estimated during the behavioral choice task). First, there was an Options screen for 4s, when the cannabis/snack and monetary options were presented; then a Choice screen, which required a response on a scale of 1–5, where 1 = strong yes for option on the left, 2 = yes for option on the left, 3 = neutral, 4 = yes for option on the right and 5 = strong yes for option on the right; then a Decision screen, which highlighted the participant’s choice; then a jittered inter-trial-interval (ITI). The left and right positioning of the puffs/snacks and money were equally distributed. The order of the trials within each run was randomized

fMRI tasks consisted of four runs of 35 choices presented in randomized order. The tasks (Fig. 1) started with an *Options* screen presenting the cannabis/snack and monetary options; followed by a *Choice* screen, requiring a 1–5 response, where 1 (strong yes) or 2 (yes) was a choice for the left option, 4 (yes) or 5 (strong yes) was a choice for the right, and 3 (neutral) was determined by a coin toss (Hare et al. 2009); followed by a *Decision* screen, which highlighted the participant’s choice; then a jittered inter-trial-interval. The decision strength ratings provided a behavioral measure of the participant’s SV for that cannabis/snack option relative to the monetary option (Hare et al. 2009, 2011), and was subsequently used for parametric modulation in analyses.

### MRI acquisition

MRI data were collected using a GE 750 3T magnet. Functional images (Blood Oxygenated Level-Dependent; BOLD) were collected from 32, 3.5 mm thick slices (0.5 mm gap) parallel to the AC/PC plane, using a T2*-sensitive spiral in-out acquisition sequence (TR = 2000msecs, TE = 27msecs, 64 × 64 matrix, 23 cm FOV, 77° flip angle) to maximize signal-to-noise ratio and minimize susceptibility artifacts in regions of interest. Voxels were 3.59 × 3.59 × 3.5 mm. A high-resolution 3D FSPGR Anatomical Sequence T1-weighted scan was also collected.

### Statistical analyses

#### Behavioral data

Data were analyzed and graphed using RStudio version 1.2.5042, SPSS version 26.0, and GraphPad Prism version 8.

We analyzed: (1) effects of cues on cannabis/snack craving (operationalized as ‘*I want cannabis*’ and ‘*I want a snack’* VAS ratings); (2) the monetary value equivalent to 3 cannabis puffs/snacks in the behavioral tasks; (3) the proportion of choices for cannabis/snacks versus money in the fMRI tasks; and (4) subjective responses to cannabis puffs and snacks during sampling sessions using repeated-measures (RM) ANOVAs or, for major departures from normality, non-parametric Wilcoxon-Signed Ranks tests.

#### fMRI Data

One participant’s fMRI data were lost; fMRI results are reported for 20 participants. Data were pre-processed using standard approaches in FSL (FMRIB Software Library, version 5.0.9, www.fmrib.ox.ac.uk/fsl) including brain extraction, motion correction (MCFLIRT), slice-timing correction, high pass temporal filtering (90-second cut-off) and spatial smoothing (5 mm full width at half maximum (FWHM) Gaussian kernel; see Supplement). Data were subject to within-subjects analysis at three levels using FSL FLAME. First-level analyses modelled brain activity within each run separately; events were modelled as boxcar functions and convolved with the canonical hemodynamic response, using the double-gamma function. Second-level analyses averaged brain activity across runs within conditions. Third-level analyses tested differences in brain activity between conditions.

Primary outcomes comprised neural SV signals for cannabis and snacks in three regions of interest (ROIs; vmPFC, VS, dPCC identified by meta-analysis) (Clithero and Rangel 2014). SV signals were statistically isolated using parametric modulation terms, within General Linear Models (GLMs), where the parametric modulator was the decision strength for that choice (rated 1–5). This identified brain areas where activity was correlated with the strength of the decision in favor of cannabis/snacks.

#### First-level analysis

We modelled Options and Choice epochs in separate GLMs. Models included the following regressors: the options/choice event, the parametrically modulated options/choice event, temporal derivatives of these regressors, six motion parameters as regressors of no interest, and a confounding regressor to scrub movement > 0.9 mm (see Supplement).

#### Second-level analyses

Fixed-effects averages from the four runs in the same condition were computed for the *Options* and *Choice* parametric modulation terms.

#### Third-level analyses

We first conducted one sample t-tests (cannabis SV > 0; snack SV > 0) to assess if cannabis and snack SV signals could be detected. We then conducted 2 × 2 RM ANOVAs, examining the interaction between and main effects of reward (cannabis, snack) and cue (active, neutral) on the neural SV signals (i.e., the parametrically-modulated terms). When the interaction and main effect non-directional F-tests were significant, we followed up with directional t-tests.

We masked third-level analyses with the ROIs and corrected for multiple comparisons within ROIs using voxelwise False Discovery Rate (FDR) correction.

This allowed us to examine, within three putatively canonical SV regions: (1) if cannabis and snack neural SV signals could be detected; (2) if SV signals differed between cannabis and snacks; and (3) how these were affected by cue exposure.

To further probe the primary analyses, we extracted the average beta values for the parametrically-modulated terms in the three ROI masks in both Options and Choice epochs and conducted 2 × 2 RM ANOVAs. We also conducted exploratory whole-brain third-level analyses.

## Results

Participants were, on average, 29 years old, with 13 years of formal education. They smoked cannabis on average 6.5 days/week (8 ‘joints’/day; see Table 2).

**Table 2:** Demographic, Mental Health and Drug Use characteristics

Demographics	*N* (%)
Sex: Female/Male	1/20 (5/95)
Race: White/Black/First Nations/Mixed	3/15/1/2 (14/71/5/10)
Ethnicity: non-Hispanic/Hispanic	17/4 (81/19)
	**Mean (S.D.) [median**,** range]**
Age (years)	29.3 (5.7) [28, 21–41]
Formal Education (Years)	12.6 (1.9) [13, 9–16]
Depression (BDI Total Score)	2.9 (4.2) [1, 0–13]
Anxiety (STICSA Total Score)	25.1 (5.9) [22, 21–42]
Trauma Exposure (TAA Total Score)	1.3 (1.3) [1, 0–5]
**Drug Use**	**N (%)**
Weekly Alcohol User	8 (38)
Daily Tobacco Cigarette Smokers	9 (43)
	**Mean (S.D.) [median**,** range]**
Cannabis Use (Days/Week)	6.5 (1.0) [7, 4–7]
Cannabis Use (‘Joints’/day)	7.7 (7.3) [5, 2–35]
Weekly Cannabis Spend (US dollars)	$97.38 (65.02) [75, 32.5–280]
SMAST^1^	2.8 (1.0) [3, 1–5]
CPQ^1^	3.9 (3.5) [3, 0–13]

*BDI = Beck Depression Inventory; STICSA = State Trait Inventory of Cognitive and Somatic Anxiety; TAA = Trauma Assessment for Adults; SMAST = Short Michigan Alcohol Screening Test; CPQ = Cannabis Problems Questionnaire.*^*1*^*One participant’s data was missing*

### Behavioral data (see supplement)

#### Effects of cues on subjective state (Fig. S3a-c)

As expected, the snack and cannabis cues increased craving for snacks and cannabis, respectively.

#### Behavioral choice task estimated indifference points for 3 cannabis puffs/snacks (Fig. S4)

There was a main effect of reward, with the estimated monetary value for 3 cannabis puffs ($1.46) greater than that for 3 snacks ($1.34). There was no significant interaction and no effect of cue (active/neutral) on indifference points.

#### Proportion of choices for cannabis/snacks versus money in the fMRI choice task

Choices for cannabis or snacks were 39–45% of total, on average, across the four conditions. There was no significant interaction, and no main effect of cue or reward on the proportion of choices for cannabis/snacks.

### fMRI results – ROI masked results

#### Options epoch

**Fig. 2:**
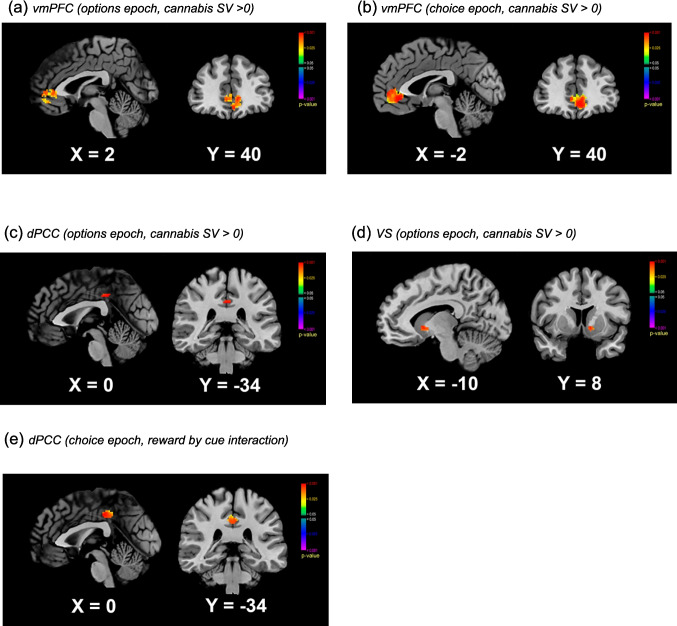
Region of interest masked, parametrically modulated fMRI results for the cannabis subjective value (SV) signal (cannabis > 0): (**a**) vmPFC (options epoch), (**b**) vmPFC (choice epoch), (**c**) dPCC (options epoch), and (**d**) VS (options epoch). (**e**) Reward by cue interaction in SV signals in dPCC (choice epoch). vmPFC = ventromedial prefrontal cortex; dPCC = dorsal posterior cingulate cortex; and VS = ventral striatum) vmPFC (options epoch, cannabis SV > 0) (b) vmPFC (choice epoch, cannabis SV > 0)

A one-sample t-test revealed cannabis SV signals in the vmPFC, dPCC and VS (Fig. 2a, c-d & Table 3a). No SV signals for snacks were observed. No main effects or interactions from the RM-ANOVA reached significance.

#### Choice epoch

In the vmPFC, a one-sample t-test revealed a significant cannabis SV signal (Fig. 2b). Furthermore, in the vmPFC, there was a main effect of reward, driven by a stronger cannabis SV than snack SV signal (Table 3b). All other tests were null.

In the dPCC, there was a significant interaction between reward and cue (Table 3b & Fig. 2e). This result was driven by a stronger cannabis than snack SV after neutral cues, with other pairwise comparisons null.

### fMRI results - extracted betas from ROIs (Fig. 3a-f)

**Fig. 3:**
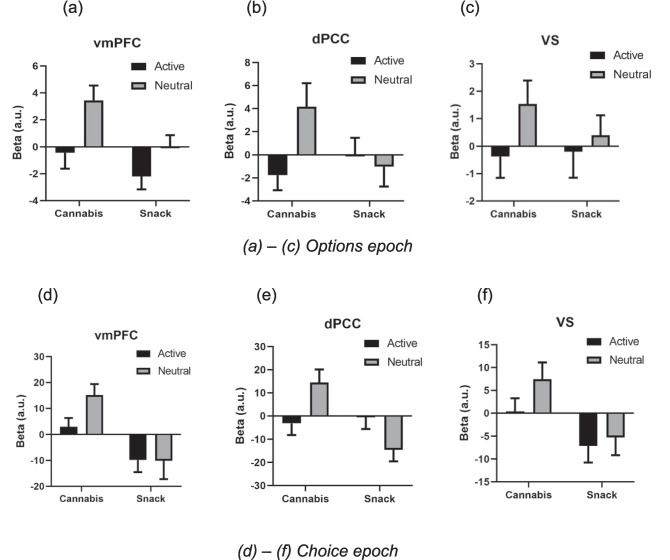
Extracted betas from regions of interest (ventromedial prefrontal cortex – vmPFC; dorsal posterior cingulate cortex – dPCC; and ventral striatum – VS) for the cannabis and snack subjective value signal in the Options epoch (**a-c**) and the Choice epoch (**d-f**). Options epoch: in the vmPFC, positive cannabis and negative snack SV signals were observed, there was a main effect of reward (cannabis > snack) and a main effect of cue (neutral > active); in the dPCC, there was a main effect of cue (neutral > active). Choice epoch: in the vmPFC, positive cannabis and negative snack SV signals were observed, and there was a main effect of reward (cannabis > snack); in the VS, a negative snack SV signal was observed and there was a main effect of reward (cannabis > snack); in the dPCC, there was an interaction between reward and cue (cannabis > snack after neutral but not active cues) and there was a main effect of reward (cannabis > snack). Extracted beta values from the general linear model have arbitrary units (a.u.) (b) (c)

#### Options epoch

In the vmPFC, a positive cannabis SV signal (t_19_ = 2.121, *p* = 0.047, Mean = 1.221, SEM = 0.576) and a negative snack SV signal were observed (t_19_ = 2.160, *p* = 0.044, Mean = -1.078, SEM = 0.499). There was a main effect of reward (F_1,19_=8.487, *p* = 0.009, η_p_^2^ = 0.309); the cannabis SV signal was greater than the snack SV signal. There was also a main effect of cue (F_1,19_=7.321, *p* = 0.014, η_p_^2^ = 0.278); SV signals following neutral cues were stronger than SV signals following active cues. The interaction term was null.

Within the dPCC, there was a main effect of cue (F_1,19_=5.043, *p* = 0.037, η_p_^2^ = 0.210); SV signals following neutral cues were stronger than SV signals following active cues. Other tests were null.

#### Choice epoch

In the vmPFC, a positive cannabis SV signal (t_19_ = 4.110, *p* = 0.001, Mean = 9.129, SEM = 2.221) and a negative snack SV signal were observed (t_19_ = 2.274, *p* = 0.035, Mean=−9.945, SEM = 4.373). There was a main effect of reward (F_1,19_=12.921, *p* = 0.002, η_p_^2^ = 0.405); cannabis SV signals were stronger than snack SV signals. Other tests were null.

In the VS, a negative snack SV signal was observed (t_19_ = 2.363, *p* = 0.029, Mean = -6.186, SEM = 2.619). The cannabis SV signal was greater than the snack SV signal (t_19_ = 2.591, *p* = 0.018).

Within the dPCC, there was an interaction between reward and cue (F_1,19_=7.853, *p* = 0.011, η_p_^2^ = 0.292), with the cannabis SV signal greater than the snack SV signal following neutral (t_19_ = 3.183, *p* = 0.005) but not active cues. Moreover, active cues reduced the positive cannabis SV signals (t_19_ = 2.380, *p* = 0.028), but increased negative snack SV signals (t_19_ = 2.203, *p* = 0.040), bringing both close to zero. There was also a main effect of reward (F_1,19_=5.865, *p* = 0.026, η_p_^2^ = 0.236); cannabis SV signals were stronger than snack SV signals.

Exploratory whole-brain results are presented in the Supplement (Figures S5, S6, S7; Tables S6, S7, S8). Of note, positive SV signals for snacks did not reach significance in whole-brain analyses.

## Discussion

SUDs can be conceptualized as pathologies of decision-making (Bickel et al. 2014; Ekhtiari et al. 2017; Redish et al. 2008). We used an experimental medicine approach to study the neural valuation of drug (cannabis) and non-drug (snack) rewards in PWUCF, following neutral and active (cannabis/snack) cues. We found positive cannabis SV signals within pre-specified ROIs (vmPFC, dPCC and VS), but the expected positive snack SV signals could not be detected. In the vmPFC and VS, cannabis SV signals were larger than snack SV signals. Additionally, within the dPCC, active cues had differential effects on cannabis and snack SV signals.

Numerous studies (Chib et al. 2009; Hare et al. 2009; Plassmann et al. 2010) and meta-analyses (Bartra et al. 2013; Clithero and Rangel 2014) have demonstrated that SV signals exist in a valuation network including the vmPFC, striatum, and dPCC. We observed cannabis SV signals in these regions, revealing that the brain’s computation of value for cannabis functions as expected in PWUCF (comparable to that for non-drug rewards in other populations). This is contrary to theories that in addiction the valuation of drugs is impaired, with decision-making habitual (Everitt and Robbins 2005). The only prior study to directly investigate neural SV signals in relation to substance use (Lawn et al. 2020) also observed expected cigarette SV signals in cigarette smokers. Moreover, three other studies – while not modelling SV encoding – found that people who use cannabis, alcohol, and tobacco recruited valuation circuitry during drug purchase decisions (Bedi et al. 2015; Gray et al. 2017; MacKillop et al. 2014). This converges with current findings to indicate that the brain’s computation of *drug* value is not “impaired” in people who frequently use drugs.

Conversely, the expected snack SV signals were not observed. Several studies have investigated value computation guiding choices for food rewards (e.g.,13,32,36); these have overwhelmingly reported SV signals for palatable food in the regions investigated. We therefore expected to observe positive snack SV signals. That these could not be detected with the fMRI measure employed – either in ROI-based or exploratory whole-brain analyses – may be indicative of dysregulation of the neural valuation of non-drug rewards in our sample. Similarly, weaker SV signals for shop vouchers (but not cigarettes) were found in the PCC of dependent compared to occasional cigarette smokers in a prior study (Lawn et al. 2020). Together, these results support theories that non-drug reward value processing may be compromised in people who frequently use cannabis or tobacco (Goldstein and Volkow 2011). In other words, the valuation system’s functionality may be moderated by reward type, such that the neural processing of drug value is intact while that of non-drug reward value is altered. Speculatively, it is possible that progressive changes to non-drug reward SV signaling plays a causal role in the pathophysiology of CUD, such that relative SV encoding for drug vs. non-drug rewards shifts during development of addiction (Hogarth and Field 2020). Future research must compare people who use cannabis on a spectrum of no, mild, moderate, and severe CUD to formally examine this.

At a behavioral level, participants interacted with snack rewards in a typical way, as they did with cannabis rewards. They were motivated to receive snacks, willing to spend money on snacks (although significantly *less* money than on cannabis, in the behavioral choice task), and they responded to snack cues with increased craving. Hence, the apparent lack of a typical SV signal for non-drug rewards warrants further investigation as a potential early marker of choice dysregulation in CUD. It is important to note that we designed the behavioral choice task to produce indifference points that equalize the number of choices for snacks/cannabis vs. money in the fMRI choice task so we could clearly probe the neural value signals, thus we did not expect, nor observe, behavioral differences in the fMRI task.

How might near-daily cannabis use itself affect valuation processes? The endocannabinoid system is thought to modulate reward circuitry (Gardner 2005; Parsons and Hurd 2015) and CB1 receptors are expressed densely in the vmPFC and VS (Curran et al., 2016). Frequent recent cannabis use could, via downregulation of the CB1 receptor (D’Souza et al. 2016), alter neural reward processing, as has been shown in some (Skumlien et al. 2021), but not all (Skumlien et al. 2022), studies of people who regularly use cannabis. However, it remains unclear why such effects would preferentially affect SV encoding for non-drug, but not drug, rewards.

Our results are concordant with literature demonstrating that people who frequently use drugs are oftentimes economically rational, and not compulsive, when they use drugs (Heyman 2013; Higgins 1997; Hogarth 2020). Although the value assigned to drugs is higher in people with SUDs (Hogarth 2020) and the SV signals potentially stronger (Lawn et al. 2020), value-based decision-making about drugs remains intact.

Findings did not support our hypothesis that active cues would preferentially augment cannabis SV signals. In the dPCC, there was an interaction between reward and cue; the cannabis SV signals were differentially affected by the presence of active cues relative to snack SV signals. The PCC is thought to be particularly sensitive to cues and craving (Brewer et al. 2013; Li et al. 2013). A similar pattern was also observed in whole-brain analyses in the angular gyrus, another region considered important in the valuation network (Clithero and Rangel 2014). This may indicate that the presence of cues weakens the relationship between encoding in some regions and subjective valuation of cannabis rewards. However, the current results do not present a clear picture regarding cue effects; further investigation into the impact of cues on drug SV encoding is therefore warranted.

As the first investigation of SV encoding in relation to cannabis use, this study had limitations. We did not include a light or non-cannabis-using control group with which to compare the non-drug SV signals of our PWUCF group. However, a wealth of evidence indicates that healthy participants show positive non-drug-reward SV signals in these ROIs (Chib et al. 2009; Clithero and Rangel 2014; Plassmann et al. 2010), including snack SV signals observed during a similar task (Hare et al. 2009). A second limitation was that cue exposure occurred outside of the scanner, with some small delays between exposure and scan initiation. This may have contributed to the relatively minor effects of the cues observed. To counter this, cues were designed to be highly potent (e.g., multisensory including engagement with personalized cannabis paraphernalia). However, future studies could valuably explore the possible impacts of value-encoding during choices for drugs in the presence of drug-paired cues, rather than after exposure. A third limitation is that we did not have sufficient power to assess the impact of factors such as gender, or variables known to be clinically-relevant markers in cannabis use, such as tobacco smoking. A fourth limitation is that we did not assess cannabis use disorder (CUD) or CUD severity. However, our participants smoked cannabis near daily, at a mean rate of 6.5 days/week, smoking an average of 7.7 joints/day. This type of high frequency, high quantity use is indicative of high risk for cannabis use disorder (Callaghan et al. 2020). Ongoing work by our team will investigate relationships between CUD severity and SV signals for cannabis and non-drug rewards.

Limitations notwithstanding, the current results suggest that this novel, controlled, multidisciplinary methodology represents a promising approach to further elucidate the neurobehavioral choice processes subserving the development and maintenance of frequent substance use. There are numerous potential avenues for future research. It remains unclear whether similar patterns of preferentially disrupted SV encoding would be observed in people using drugs other than cannabis and cigarettes (Lawn et al. 2020). Similarly, the effects of state factors like withdrawal on SV encoding for drug and non-drug reinforcers in different populations remains unknown. A key question for future investigation will be the extent to which these neural SVs change following cessation of drug use. Links between neural SV signals, behavior, and clinical outcomes should be investigated.

This was the first study to investigate neural SV signals for a real, tangible, illicit drug: other studies of drug-related choice used hypothetical rewards (Hogarth 2020) or drug images (Moeller et al. 2013) or studied legal drugs (Lawn et al. 2020; MacKillop et al. 2014). In PWUCF, cannabis SV signals were intact, while snack SV signals could not be detected, tentatively suggesting dysregulation of these neurobiological choice processes.

## Supplementary Information

Below is the link to the electronic supplementary material.

ESM 1 (ODT 2.71 MB)
