# Supplementary material for: Value signals guiding choices for cannabis versus non-drug rewards in people who use cannabis near-daily
Source: Psychopharmacology (Berl). 2025 Feb 10;242(4):681–91. doi: 10.1007/s00213-025-06746-6 (PMC11890362; doi:10.1007/s00213-025-06746-6)
Supplement: Supplementary file 1 [file 213_2025_6746_MOESM1_ESM.odt]

Value signals guiding choices for cannabis versus non-drug rewards in regular cannabis users

SUPPLEMENT

Will Lawna,b, Xuejun Haoc, Anna B. Konovad, Margaret Haneyc, Ziva D. Coopere, Nicholas Van Damf, Paul Glimcherg, Gillinder Bedih*

a Department of Psychology, Institute of Psychiatry Psychology and Neuroscience, King’s College London, London, UK

b Clinical Psychopharmacology Unit, University College London, London, UK

c Department of Psychiatry, Columbia University Medical Center and New York State Psychiatric Institute, NY, USA

d Department of Psychiatry, Rutgers University Behavioral Health Care and the Brain Health Institute, Rutgers University, Piscataway, NJ, USA

e UCLA Center for Cannabis and Cannabinoids, Jane and Terry Semel Institute for Neuroscience and Human Behavior, Department of Psychiatry and Biobehavioral Sciences, David Geffen School of Medicine, University of California, Los Angeles

f Contemplative Studies Centre, Melbourne School of Psychological Sciences, University of Melbourne, Melbourne, Australia. 

g Institute for the Interdisciplinary Study of Decision Making, New York University, NY, USA

h Centre for Youth Mental Health, University of Melbourne, and Orygen, Melbourne, Australia

Contents

[Methods	3](#__RefHeading__2763_362567862)

[Participants	3](#__RefHeading__2765_362567862)

[Measures 	3](#__RefHeading__2767_362567862)

[Demographic assessments	3](#__RefHeading__2769_362567862)

[Mental health assessments	3](#__RefHeading__2771_362567862)

[Drug use assessments	3](#__RefHeading__2773_362567862)

[Other baseline assessments	3](#__RefHeading__2775_362567862)

[Choice tasks	3](#__RefHeading__2777_362567862)

[Subjective Mood State 	4](#__RefHeading__2779_362567862)

[Experimental protocol 	5](#__RefHeading__2781_362567862)

[Screening	5](#__RefHeading__2783_362567862)

[Choice days (days 1, 2, 5, & 6)	5](#__RefHeading__2785_362567862)

[Days 3 & 4 (no choices)	5](#__RefHeading__2787_362567862)

[Cannabis and snacks	5](#__RefHeading__2789_362567862)

[Cues	5](#__RefHeading__2791_362567862)

[Statistical analyses	6](#__RefHeading__2793_362567862)

[Behavioral Data	6](#__RefHeading__2795_362567862)

[fMRI Data	7](#__RefHeading__2797_362567862)

[Results	9](#__RefHeading__2799_362567862)

[Behavioral results	9](#__RefHeading__2801_362567862)

[Whole brain fMRI results (unmasked)	9](#__RefHeading__2803_362567862)

[Tables	11](#__RefHeading__2805_362567862)

[Table S1. Representative study events timeline for choice days (days 1,2, 5 and 6)	11](#__RefHeading__2807_362567862)

[Table S2. Sampling session Visual Analogue Scale parametric results	13](#__RefHeading__2809_362567862)

[Table S3. Sampling session Visual Analogue Scale non-parametric results	14](#__RefHeading__2811_362567862)

[Table S4. Masked fMRI follow-up after main effect of drug: Choice>Snack (Choice Epoch, vmPFC)	15](#__RefHeading__2813_362567862)

[Table S5. Masked fMRI follow-up after reward x cue interaction: Cannabis-neutral>Snack-neutral (Choice Epoch, dPCC)	15](#__RefHeading__2815_362567862)

[Table S6. Whole brain unmasked analysis of Cannabis > 0 (Options Epoch)	16](#__RefHeading__2817_362567862)

[Table S7. Whole brain unmasked analysis of reward x cue interaction (Choice Epoch)	17](#__RefHeading__2819_362567862)

[Table S8. Whole brain unmasked analysis for t-test of Cannabis > Snack (Choice Epoch)	18](#__RefHeading__2821_362567862)

[Table S9. CONSORT 2010 checklist of information to include when reporting a randomized trial	19](#__RefHeading__2823_362567862)

[Figures	21](#__RefHeading__2825_362567862)

[Figure S1. Masks	21](#__RefHeading__2827_362567862)

[Figure S2. Subjective mood state during cannabis and snack sampling sessions (Visual Analogue Scales)	22](#__RefHeading__2829_362567862)

[Figure S3a. ‘Aroused’ ratings during cue sessions 	24](#__RefHeading__2831_362567862)

[Figure S3b. ‘Want snack’ ratings during cue sessions	24](#__RefHeading__2833_362567862)

[Figure S3c. ‘Want cannabis’ ratings	25](#__RefHeading__2835_362567862)

[Figure S4. Mean indifference points from behavioral choice task	26](#__RefHeading__2837_362567862)

[Figure S5. One sample t-test of Cannabis > 0 across the whole brain (Options epoch)	27](#__RefHeading__2839_362567862)

[Figure S6. Reward X cue interaction across the whole brain (Choice epoch)	27](#__RefHeading__2841_362567862)

[Figure S7. Cannabis > Snack across the whole brain (Choice epoch)	28](#__RefHeading__2843_362567862)

[Figure S8. Distribution of participants 	29](#__RefHeading__2845_362567862)

[References	30](#__RefHeading__2847_362567862)

# Methods

## Participants

Inclusion criteria were: (1) smokes cannabis ≥4 days/week (self-report and positive THC urine toxicology); (2) smokes ≥2 cannabis cigarettes/day-of-use; (3) aged 21-50 years old; (4) right-handed; and (5) using effective contraception (if female). Exclusion criteria were: (1) uses illicit drugs other than cannabis (>2 days/week); (2) regularly smokes cannabis in forms other than ‘blunts’ or ‘joints’; (3) requesting drug treatment; (4) current DSM-IV Axis I psychiatric disorder (First et al., 2004) requiring medical intervention; (5) contraindicated medical illness or medications; (6) contraindication for Magnetic Resonance Imaging (MRI); (7) clinically-significant dietary restraint (Stunkard and Messick, 1985); and (8) pregnant. Our participants reported no use of any other illicit drugs in the past month (opioids, cocaine, amphetamines, MDMA).

## Measures 

### Demographic assessments

Educational level was measured as years spent in formal education. Race was self-reported as one of the following categories: Caucasian, African American, American Indian or Alaska Native, or Mixed. Ethnicity was self-reported as Hispanic or non-Hispanic.

### Mental health assessments

During screening, Axis 1 psychiatric disorder based on the Diagnostic and Statistical Manual of Mental Disorders (DSM-IV) (First et al., 2004) was assessed with the Structured Clinical Interview for DSM-IV (SCID-IV) (Ventura et al., 1998). Depression symptoms were measured with the Beck Depression Inventory (Beck et al., 1996) total score. Anxiety symptoms were measured using the State-Trait Inventory for Cognitive and Somatic Anxiety (Ree et al., 2000) total score. Exposure to traumatic events was measured using the Trauma Assessment for Adults (Resnick et al., 1993) total score. 

### Drug use assessments

Cannabis use was quantified via structured interview as: days/week of cannabis consumption, ‘joints’/day on days when cannabis was used, and amount spent weekly on cannabis (USD). Alcohol consumption and tobacco smoking were measured with days/week of use. Problematic cannabis consumption was measured using the 21-item Cannabis Problems Questionnaire (Copeland et al., 2005). Problematic alcohol consumption was measured using the Short Michigan Alcohol Screening Test (Selzer et al., 1975). Recent drug use was assessed using urine drug screens (5-Panel drug screen, All Test North America, Gilbert, AZ), alcohol breathalyzer tests (Select S80, BACTrack, San Francisco, CA), and a carbon monoxide monitor (Vitalgraph, Lenexa, KS).

### Other baseline assessments

Biologically female participants were tested for pregnancy using a urine pregnancy test at screening and admission (Alere hCG Dipstick, Alere, Orlando, FL). All participants completed clinical blood tests and electrocardiogram during screening to assess for contraindicating medical conditions. 

### Choice tasks

Behavioral choice tasks

A behavioral choice task was completed on each choice day before the fMRI scan. The purpose of this task was to estimate that participant’s monetary value (i.e., the indifference price point) for 3 cannabis puffs or 3 snacks (dependent on condition) on that day. This represented the dollar value at which the participant was equally likely to choose 3 puffs/snacks or money. It was calculated using a logistic regression method (Maier et al., 2015; Tom et al., 2007). This value was then entered into the fMRI task as the stable monetary alternative (Hare et al., 2009). The value for 3 puffs/snacks was chosen as the midpoint between the total number of puffs/snacks available in the fMRI task (which had a range of 0-6 puffs/snacks), so that participants would vary their choices in the scanner and choice behavior between conditions would be approximately normalized. This aimed to allow us to isolate SV neural activity from behavior, such that differences could be attributed to encoding, rather than behavioral differences (Hare et al., 2009). Each condition focused on either cannabis or snack decisions (not both), so that we could isolate the SV signal for cannabis or snacks, relative to the common currency of money, without disruption from the other reward. 

In the behavioral choice task, participants made 30 choices between 0-6 cannabis puffs/snacks (dependent on condition) and monetary values from $0 to $30. One choice from the behavioral choice task was randomly selected and implemented during the first administration session that afternoon i.e., if the item randomly selected was a decision between $5 and 3 puffs and the participant chose puffs, they would receive 3 puffs of cannabis in the first administration session that afternoon. If they chose money in the selected decision, they received $5 cash (added to their study payment) after the scan and remained in the laboratory without undergoing the first administration session. 

fMRI Choice Task (Hare et al., 2009)

Participants repeatedly chose between 0-6 cannabis puffs/snacks (dependent on condition) and a fixed monetary alternative (the indifference point estimated in the behavioral choice task). The fMRI choice task consisted of four runs of 35 choices each, making a total of 140 choices; each run lasted for 8 mins 22 secs (see Figure 1).

Using the individualized indifference point for 3 puffs/snacks as the fixed alternative aimed to result in participants choosing cannabis/snacks when there were 4, 5, or 6 puffs/snacks available, and the monetary option when there were 0, 1, or 2 puffs/snacks available, with choices for 3 puffs/snacks versus the monetary option equally likely. In 22 of 80 scans, the initial reference option (indifference point) estimated by the behavioral task did not result in variable choices during the first run; in those cases, an increased or decreased monetary option was entered for runs 2-4 to increase response variability and only those runs were used for analyses.

One choice from the fMRI task was randomly selected and implemented during the second administration session that afternoon as follows: 1 and 2 responses received the item on the left, 4 and 5 responses received the item on the right, and 3 (neutral) responses resulted in a coin flip to determine which option was received. If the participant chose money in the selected decision, they received that amount of money (added to their study payment) after the scan and remained in the laboratory without undergoing the second administration session. 

Second behavioral choice task 

There was a second behavioral task, which enabled examination of the economic rationality of choices in this population; these data will be reported separately. 

### Subjective Mood State 

Subjective mood state in response to the reinforcers (cannabis and snacks) and the cues was measured with Visual Analogue Scales (VAS). The VAS (Bedi et al., 2013) employed during sampling and administration sessions comprised 35 items (rated from 0 to 100 on a 100mm computer-presented scale) that were grouped together into seven cluster scales by calculating the arithmetic means of individual items. These seven clusters (and individual items) were: high (good drug effect & high), miserable (depressed, irritable & miserable), tired (clumsy, sedated, sleepy, withdrawn, unmotivated & tired), on edge (anxious, jittery, restless, stimulated & on edge), bad effect (blurred vision, chills, dizzy, headache, heart pound, nauseous, stomach pain, upset stomach & bad drug effect), content (alert, energetic, mellow, self-confident, social, talkative & content), and confused (can’t concentrate, forgetful & confused). We also collected VAS data for I want cannabis and I want a snack.

Subjective responses to cues were assessed with 3 VAS measuring ratings of I want cannabis, I want a snack and aroused. 

## Experimental protocol 

### Screening

Volunteers underwent comprehensive examination including electrocardiogram, blood/urine analysis, physical examination, psychiatric assessment, urine toxicology, alcohol breathalyzer, carbon monoxide, and pregnancy tests.

### Choice days (days 1, 2, 5, & 6)

Participants were provided with their selected breakfast. The same breakfast, lunch and dinner was provided on each choice day. Participants were allowed to eat unlimited servings, however all meals on scanning days had low salt, fat, and sugar content to ensure that the snacks available were reinforcing. After breakfast, lunch, and during the afternoon administration sessions, cigarette smokers were allowed to smoke a cigarette to prevent nicotine withdrawal (Haney et al., 2013). Participants ate dinner at 5.30pm before being returned to the inpatient unit. Nothing other than water was consumed after dinner until the following day. 

### Days 3 & 4 (no choices)

Days 3 and 4 comprised weekend days. On these days, participants underwent a single experimenter-administered cannabis session, receiving six cannabis puffs. This ensured that no participant went more than two days without cannabis, limiting effects of cannabis withdrawal on choices. Participants ate dinner at 5.30pm before returning to the inpatient unit. Nothing other than water was consumed after dinner until the following day.

## Cannabis and snacks

Cannabis (5.6% ∆9- tetrahydrocannabinol; <0.1% cannabidiol) cigarettes were provided by the National Institute for Drug Abuse (NIDA). The cannabis cigarettes contain approximately 800mg of cannabis. The cannabis is 5.6% THC. Each cigarette contains approximately 44.8mg of THC (~9 standard THC units, at 5mg/unit). Each cigarette is consumed in approximately 6 puffs and each puff takes about 5 seconds. Thus, each 5 second puff contains approximately 7.5mg THC (1.5 standard THC units, at 5mg/unit).

 Vital signs were monitored throughout sampling and administration sessions for safety. Cannabis was well-tolerated and did not produce changes in vital signs that required medical attention. 

During training, participants were given a list of 38 snacks including hot (e.g., fries), cold (e.g., chocolate), and frozen (e.g., ice pops) options, all between 50 and 100 calories. They selected their 10 favorite snacks from the list, which were then stocked prior to their inpatient admission. During snack sessions, participants could choose to eat any of the 10 snacks they had previously selected. 

## Cues

Different cues were used for the behavioral and fMRI tasks to avoid habituation. The visual cues preceding the behavioral task were presented on the laptop screen for 10 seconds each (total – 5 mins). Cannabis and snack cues were customized for this study; neutral cues were selected from the International Affective Pictures System (Lang et al., 1997). Visual cues were presented immediately before the behavioral choice task.

For multisensory cannabis cues, preceding the fMRI task, participants were given approximately 1.5 grams of active cannabis with their preferred ‘blunt’ or ‘joint’ papers and asked to roll a blunt/joint and then hold and smell it. For multisensory snack cues, they were provided with samples of their 10 chosen snacks (heated if appropriate) and asked to unwrap, smell, and touch the snacks. For the two neutral cue sessions, participants touched and smelled one of two differently scented candles prior to wrapping the candle as a gift. Immediately following cues, the participant was taken into the scanning room and the was scan started. During initial localizer scans and between choice task runs, participants viewed more cue pictures and listened to auditory stimuli according to that day’s condition to facilitate continuity of cue effects during scanning. Auditory stimuli were customized for this study and consisted of people smoking/talking about cannabis (cannabis cues), eating/talking about food (snack cues), and background noise/talking (neutral cues).

No cue stimuli were repeated. We used visual cues before the behavioral economic task to minimize habituation effects that might have occurred had we used the multisensory cues twice.

Before and after both the visual and the multisensory cues, participants were asked: ‘RIGHT NOW do you want cannabis/a snack?’ and ‘RIGHT NOW are you aroused?’. Scales were rated from ‘Not at all’ (0) to ‘A lot’ (100).

## Statistical analyses

### Behavioral Data

When data met relevant assumptions, they were analyzed using repeated-measures (RM) ANOVA. RM-ANOVA used within-subjects factors of reward (cannabis, snack) and cue (active, neutral), and, depending on the analysis, time. The Greenhouse-Geisser correction was employed when Mauchley’s test of sphericity indicated departure from sphericity. Alpha was set at 0.05 for the omnibus ANOVAs. Significant main effects and interactions were followed up with local Bonferroni-corrected t-tests. When data did not meet relevant assumptions, non-parametric Wilcoxon-Signed Ranks tests were conducted, with manually Bonferroni-adjusted alpha thresholds.

All behavioral data apart from demographic and drug use data were subject to winsorizing. Outliers were identified using Tukeys’ method as data points that were greater than three times the interquartile range (IQR) from the lower quartile or upper quartile. These outliers were replaced with the closest non-outlying data point. Data were holistically examined for normality using: visual inspection of histograms; skewness and kurtosis scores >1; and Kolmogrov-Smirnov tests.

Graphs were produced using winsorized data. All data are graphed using means and standard errors, even if data were non-normal.

Missing data were left missing in a pairwise fashion and not imputed. Missing behavioral data are highlighted in the relevant Results sub-sections.

Subjective effects from sampling sessions (Figure S2)

The following clusters/variables were analyzed with RM ANOVA with within-subjects factors of time (1-8) and reward (cannabis, snack): tired, on edge, content and want cannabis. The following clusters were analyzed with Wilcoxon-Signed Rank tests due to substantive departures from normality: high, miserable, bad effect and confused. Within these four clusters, several outliers that were winsorized. Four participants were excluded from these analyses due to incomplete datasets (leaving n=17).

Subjective effects of cues (Figure S3a-c) 

For want cannabis, there was a substantive departure from normality, so we used non-parametric Wilcoxon-Signed Ranks tests. We compared pre-visual and post-multisensory cues want cannabis ratings within each cue day separately. Four outliers were winsorized in the post-multisensory cannabis-active condition. Data were missing for one participant (n=20).

For aroused, we conducted a RM ANOVA with factors of time (pre-visual cues, post-visual cues, pre-multisensory cues, post-multisensory cues), cue (neutral, active) and reward (cannabis, snack). For want snack we conducted a RM ANOVA with factors of time and cue, for the snack days only.

Indifference points and proportions of choices for cannabis/snacks in behavioral and fMRI choice tasks

For both indifference points and the proportion of choices for cannabis/snacks in the behavioral and fMRI choice tasks, we used RM ANOVA with within-subjects factors of reward (cannabis, snack) and cue (active, neutral).

### fMRI Data

One participant’s fMRI data was lost, leaving n=20.

Pre-processing

Pre-processing was carried out using FSL (FMRIB Software Library, version 5.0.9, www.fmrib.ox.ac.uk/fsl) as in a previous study (Bedi et al., 2019). For quality control, the raw fMRI data were first inspected to detect scanner or intensity inconsistencies, and to detect any obvious movement; next translational and rotational movement were checked for each run of data; and the two-step registration process was checked. Data were all acceptable, specifically no run of data showed a maximum movement of greater than 3.5mm (the size of one dimension of the voxel).

Standard pre-processing was applied, including brain extraction, motion correction (MCFLIRT), slice-timing correction, high pass temporal filtering (90-second cut-off) and spatial smoothing (5 mm full width at half maximum (FWHM) Gaussian kernel). The anatomical images were subject to inhomogeneity correction using N4 bias field correction processes (ANT, [http://picsl.upenn.edu/software/ants/](http://picsl.upenn.edu/software/ants/)) and FSL’s Brain Extraction Tool (BET) process. Manual adjustments were made if required, to ensure that the brain was accurately extracted.

Masking using Regions of Interest (ROI) (Figure S1)

Previous meta-analysis(Clithero and Rangel, 2014) showed the vmPFC had three centers of activity: [-2, 40, -6], [4, 30, -16], [-4, 60, -10]; the ventral striatum had two centers of activity: [-10, 8, -6] and [10, 12, -10]; and the dPCC had one center of activity: [0, -34, 42]. Spheres were formed around these centers and combined to create three separate masks. The average diameter of the vmPFC spheres was 21mm, the average diameter of the ventral striatum was 15mm, and the dPCC sphere had a diameter of 15mm. These were used as masks for the primary analyses.

First level analyses

The confounding regressor was used to scrub volumes exceeding a framewise displacement threshold of 0.9mm. The events were modelled as boxcar functions with the whole duration of the event and convolved with the canonical hemodynamic response, using the double-gamma function.

Secondary ROI analyses 

We conducted secondary analyses in which we extracted average betas for SV signals from the ROIs described above. We analyzed the extracted betas using 2x2 RM ANOVAs. Extracted betas were winsorized before analysis.

Whole brain analyses without masking

For hypotheses generation for future studies, we conducted exploratory whole-brain analyses using the same 2X2 RM ANOVAs, but without masking the third-level analyses with ROIs. We used a cluster-defining threshold of z=2.3 and a corrected cluster extent threshold of p<0.05 using FWE. 

# Results

## Behavioral results

Subjective mood state during the sampling sessions (Tables S2 and S3, Figure S2)

Of all eight variables, only high was affected during the sampling sessions. At baseline – before sampling commenced – there was no difference in high ratings between cannabis and snack sessions. At every other timepoint, high ratings were greater during cannabis than snack (ps<0.001).

Subjective effects of cues (Figure S3a-c) 

For want cannabis, data were missing for one participant (n=20). On the active cue day, want cannabis was greater at post-multisensory cues compared to pre-visual cues (Z=3.062, p=0.002), but on the neutral cue day this was not the case (Z=1.060, p=0.289). 

For want snack, there was a significant interaction between cue and time (F3,60=4.505, p=0.012, ηp2=0.184) and a main effect of time (F3,60=5.321, p=0.007, ηp2=0.210). On the active cue day, want snack was higher at post-multisensory cues compared to post-visual cues (t20=3.250, p=0.024) and to the pre-multisensory cues (t20=3.584, p=0.011), but there were no significant differences on the neutral cue day. The main effect of time was driven by greater want snack ratings at post-multisensory cues compared to pre-multisensory cues (t20=3.559, p=0.012).

For aroused, data were missing for one participant (n=20). The only significant effect was time (F3,57=4.181, p=0.019, ηp2=0.180). All other terms were non-significant (ps>0.095). Post-hoc t-tests showed that, compared to post-multisensory cues, all other timepoints had non-significantly lower aroused levels (pre-visual vs. post-multi: t19=2.495, p=0.132; post-visual vs. post-multi: t19=2.554, p=0.116; pre-multi vs. post-multi: t19=2.585, p=0.115).

This demonstrates that the cues increased craving for snacks and cues (operationalized as ratings of want cannabis and want a snack), with no effect on subjective arousal. 

Indifference point in the behavioral choice task (Figure S4).

We conducted an RM-ANOVA with factors of reward and cue. There was no interaction between reward and cue (F1, 20=1.452, p=0.242, ηp2=0.068). There was no main effect of cue (F1, 20=1.164, p=0.294, ηp2=0.055). There was a main effect of reward (F1, 20=4.425, p=0.048, ηp2=0.181), with the indifference point for cannabis (Mean=USD1.46, SEM=0.229) higher than that for snacks (Mean=USD1.34, SEM=0.219).

Proportion of choices for cannabis/snack (vs. money) during fMRI task 

The average proportion of choices for cannabis or snacks were similar in each condition. Following active cues, cannabis was chosen 45.0% (SD=12.0) of the time, and following neutral cues, cannabis was chosen 40.7% (SD=16.7) of the time. Following active cues, snacks were chosen 41.8% (SD=11.0) of the time, and following neutral cues, snacks were chosen 39.4% (SD=18.8) of the time. There was no significant interaction between reward and cue (F1,19=0.097, p=0.758, ηp2=0.005) and no significant effect of cue (F1,19=2.588, p=0.124, ηp2=0.120) or reward (F1,19=0.370, p=0.550, ηp2=0.019). This demonstrates that the individualized indifference points worked in standardizing the proportion of choices for cannabis/snacks in all conditions during the fMRI choice task.

## Whole brain fMRI results (unmasked)

Options Epoch 

One-sample t-tests showed there were cannabis SV signals in the superior frontal gyrus and occipital regions.

Choices Epoch

There was an interaction between reward and cue in the right angular gyrus. This was driven by more negative snack SV signals following active cues than neutral cues, but more positive cannabis SV signals following neutral cues than active cues.

There was a main effect of reward, with cannabis value signals stronger than snack value signals, in regions with peaks in the superior frontal gyrus, anterior cingulate gyrus and superior parietal lobule.

# Tables

### Table S1. Representative study events timeline for choice days (days 1,2, 5 and 6)

| Time | Event |
| --- | --- |
| 08:00 | Meal |
| 08:45 | Pre-visual cues VAS |
| 08:50 | Visual cues & post-visual cues VAS |
| 09:00 | Behavioral choice task |
| 09:45 | Pre-multisensory cues VAS |
| 09:50 | Multisensory cues & post-multisensory cues VAS |
| 10:00 | fMRI choice task |
| 11:00 | Meal |
| 11:30 | Administration session 1 (rewards earned from behavioral choice task) |
| 15:00 | Administration session 2 (rewards earned from fMRI choice task) |
| 17:30 | Meal |

### Table S2. Sampling session Visual Analogue Scale parametric results

Subjective effects of sampling cannabis and snacks. RM-ANOVA results for parametric data: tired, on edge, content and want cannabis, during snack and cannabis administration. F test statistics, p values and partial eta squared effect sizes are shown for main effects of time and reward, and their interaction. 

|  | Time |  |  | Reward |  |  | Time x Reward interaction |
| --- | --- | --- | --- | --- | --- | --- | --- |
|  | F | p | ηp2 | F | p | ηp2 | F | p | ηp2 |
| Tired | 0.507 | 0.828 | 0.031 | 0.108 | 0.746 | 0.007 | 0.108 | 0.746 | 0.007 |
| On Edge | 2.440 | 0.064 | 0.132 | 0.352 | 0.562 | 0.014 | 0.679 | 0.0585 | 0.041 |
| Content | 1.197 | 0.321 | 0.070 | 0.221 | 0.645 | 0.014 | 0.679 | 0.0585 | 0.041 |
| Want Cannabis | 2.299 | 0.124 | 0.126 | 0.099 | 0.757 | 0.006 | 1.704 | 0.160 | 0.096 |
|  |  |  |  |  |  |  |  |  |  |

### Table S3. Sampling session Visual Analogue Scale non-parametric results

Subjective effects of sampling cannabis and snacks. Cannabis vs. snack Wilcoxon Signed Rank tests: high, miserable, bad effect and confused. Z test statistics and uncorrected p values are shown for cannabis vs. snack differences. The alpha threshold is reduced to 0.006 to correct for eight tests per scale.

|  | T1 | T2 | T3 | T4 | T5 | T6 | T7 | T8 |
| --- | --- | --- | --- | --- | --- | --- | --- | --- |
| High | Z=0, p=1 | Z=3.516, p<0.001 | Z=3.621, p<0.001 | Z=3.622, p<0.001 | Z=3.621, p<0.001 | Z=3.624, p<0.001 | Z=3.622, p<0.001 | Z=3.621, p<0.001 |
| Miserable | z=-2.33, p=0.02 | Z=2.049, p=0.040 | Z=2.060, p=0.039 | Z=1.693, p=0.090 | Z=2.060, p=0.039 | Z=0, p=1 | Z=2.121, p=0.034 | Z=0.535, p=0.593 |
| Bad effect | Z=0.943, p=0.345 | Z=1.648, p=0.099 | Z=0.471, p=0.638 | Z=0.629, p=0.529 | Z=0.044, p=0.965 | Z=0.624, p=0.533 | Z=0.654, p=0.513 | Z=1.960, p=0.050 |
| Confused | Z=2.539, p=0.011 | Z=0.356, p=0.722 | Z=0, p=1 | Z=0, p=1 | Z=0, p=1 | Z=1.840, p=0.066 | Z=2.207, p=0.027 | Z=0, p=1 |

### Table S4. Masked fMRI follow-up after main effect of drug: Choice>Snack (Choice Epoch, vmPFC)

| Contrast | Region | Cluster Size | Center Coordinates (MNI) | Max. p value | Max. Coordinates (MNI) |
| --- | --- | --- | --- | --- | --- |
| Cannabis > Snack | vmPFC | 770 voxels | -3, 41, -5 | 0.00005 | -2, 34, -8 |

### Table S5. Masked fMRI follow-up after reward x cue interaction: Cannabis-neutral>Snack-neutral (Choice Epoch, dPCC)

| Cannabis-Neutral > Snack-Neutral: Choice Epoch |
| --- |
| Region | Cluster Size | Center Coordinates (MNI) | Max. p value | Max. Coordinates (MNI) |
| dPCC | 168 voxels | 0, -34, 41 | 0.0002 | 0, -28, 38 |

### Table S6. Whole brain unmasked analysis of Cannabis > 0 (Options Epoch)

These results are visualized in figure S5.

| Cluster index | Cluster Size | Cluster P Value | Center Coordinates (MNI) | Center Coordinates label | Max. p value | Max. Coordinates (MNI) |
| --- | --- | --- | --- | --- | --- | --- |
| 1 | 2691 voxels | 6x10-8 | -1 -53 23 | Superior frontal gyrus | 7x10-7 | 2 -62 18 |
| 2 | 1048 voxels | 0.001 | -24 -92 -12 | Occipital pole | 1x10-7 | -20 -90 -16 |
| 3 | 733 voxels | 0.012 | 32 -88 -12 | Lateral occipital cortex, inferior | 0.00002 | 44 -82 -8 |
| 4 | 671 voxels | 0.019 | -46 -74 38 | Lateral occipital cortex, superior | 0.0002 | -52 -76 34 |

### Table S7. Whole brain unmasked analysis of reward x cue interaction (Choice Epoch)

These results are visualized in figure S6.

| Cluster index | Cluster Size | Cluster P Value | Center Coordinates (MNI) | Center coordinates label | Max. p value | Max. Coordinates (MNI) |
| --- | --- | --- | --- | --- | --- | --- |
| 1 | 598 voxels | 0.03 | 45 -53 52 | Right angular gyrus | 0.0003 | 50 -46 42 |

### Table S8. Whole brain unmasked analysis for t-test of Cannabis > Snack (Choice Epoch)

These results are visualized in figure S7.

| Cluster index | Cluster Size | Cluster P Value | Center Coordinates (MNI) | Center coordinates label | Max. p value | Max. Coordinates (MNI) |
| --- | --- | --- | --- | --- | --- | --- |
| 1 | 1346 voxels | 2x10-6 | -10 -9 63 | Superior frontal gyrus | 0.0001 | -22 8 64 |
| 2 | 1311 voxels | 3x10-6 | 0 43 4 | Anterior cingulate gyrus | 0.00002 | 4 40 8 |
| 3 | 670 voxels | 0.0003 | 26 -40 63 | Superior parietal lobule | 0.00002 | 32 -46 56 |

### Table S9. CONSORT 2010 checklist of information to include when reporting a randomized trial

| Section/Topic | Item No | Checklist item | Reported on page No |
| --- | --- | --- | --- |
| Title and abstract |
|  | 1a | Identification as a randomised trial in the title | N/A |
| 1b | Structured summary of trial design, methods, results, and conclusions (for specific guidance see CONSORT for abstracts) | 2 |
| Introduction |
| Background and objectives | 2a | Scientific background and explanation of rationale | 3-5 |
| 2b | Specific objectives or hypotheses | 5 |
| Methods |
| Trial design | 3a | Description of trial design (such as parallel, factorial) including allocation ratio | 6 |
| 3b | Important changes to methods after trial commencement (such as eligibility criteria), with reasons | N/A |
| Participants | 4a | Eligibility criteria for participants | 6/S3 |
| 4b | Settings and locations where the data were collected | 6 |
| Interventions | 5 | The interventions for each group with sufficient details to allow replication, including how and when they were actually administered | 6-9/S3-S5 |
| Outcomes | 6a | Completely defined pre-specified primary and secondary outcome measures, including how and when they were assessed | 10-11 |
| 6b | Any changes to trial outcomes after the trial commenced, with reasons | N/A |
| Sample size | 7a | How sample size was determined | S3 |
| 7b | When applicable, explanation of any interim analyses and stopping guidelines | N/A |
| Randomisation: |  |  |  |
| Sequence generation | 8a | Method used to generate the random allocation sequence | 6 |
| 8b | Type of randomisation; details of any restriction (such as blocking and block size) | 6 |
| Allocation concealment mechanism | 9 | Mechanism used to implement the random allocation sequence (such as sequentially numbered containers), describing any steps taken to conceal the sequence until interventions were assigned | N/A |
| Implementation | 10 | Who generated the random allocation sequence, who enrolled participants, and who assigned participants to interventions | 6 |
| Blinding | 11a | If done, who was blinded after assignment to interventions (for example, participants, care providers, those assessing outcomes) and how | N/A |
| 11b | If relevant, description of the similarity of interventions | N/A |
| Statistical methods | 12a | Statistical methods used to compare groups for primary and secondary outcomes | 9-11; S6-7 |
| 12b | Methods for additional analyses, such as subgroup analyses and adjusted analyses | 9-11; S6-7 |
| Results |
| Participant flow (a diagram is strongly recommended) | 13a | For each group, the numbers of participants who were randomly assigned, received intended treatment, and were analysed for the primary outcome | 10; S18 |
| 13b | For each group, losses and exclusions after randomisation, together with reasons | S18 |
| Recruitment | 14a | Dates defining the periods of recruitment and follow-up | S3 |
| 14b | Why the trial ended or was stopped | N/A |
| Baseline data | 15 | A table showing baseline demographic and clinical characteristics for each group | Table 2 |
| Numbers analysed | 16 | For each group, number of participants (denominator) included in each analysis and whether the analysis was by original assigned groups | S18 |
| Outcomes and estimation | 17a | For each primary and secondary outcome, results for each group, and the estimated effect size and its precision (such as 95% confidence interval) | 12-14 |
| 17b | For binary outcomes, presentation of both absolute and relative effect sizes is recommended | N/A |
| Ancillary analyses | 18 | Results of any other analyses performed, including subgroup analyses and adjusted analyses, distinguishing pre-specified from exploratory | N/A |
| Harms | 19 | All important harms or unintended effects in each group (for specific guidance see CONSORT for harms) | N/A |
| Discussion |
| Limitations | 20 | Trial limitations, addressing sources of potential bias, imprecision, and, if relevant, multiplicity of analyses | 17-18 |
| Generalisability | 21 | Generalisability (external validity, applicability) of the trial findings | 18 |
| Interpretation | 22 | Interpretation consistent with results, balancing benefits and harms, and considering other relevant evidence | 15-18 |
| Other information |  |
| Registration | 23 | Registration number and name of trial registry | N/A |
| Protocol | 24 | Where the full trial protocol can be accessed, if available | N/A |
| Funding | 25 | Sources of funding and other support (such as supply of drugs), role of funders | 19 |

# Figures

### Figure S1. Masks

Ventromedial prefrontal cortex (vmPFC) shown in yellow centered at [-2, 40, -6], [4, 30, -16], [-4, 60, -10]; bilateral ventral striatum shown in green centered at [-10, 8, -6] & [10, 12, -10]; and dorsal posterior cingulate cortex (dPCC) centered at [0, -34, 42] shown in blue. The initial half of images from top left are right hemisphere and the second half of the images are the left hemisphere.

### Figure S2. Subjective mood state during cannabis and snack sampling sessions (Visual Analogue Scales)

Data are means and standard errors. Timepoints from 2-7 occurred 4 minutes after a cannabis puff or snack. 

### Figure S3a. ‘Aroused’ ratings during cue sessions 

Effects of cues (active, neutral) and reward (snack, cannabis) at the four timepoints (pre-visual cues, post-visual cues, pre-multisensory cues, post-multisensory cues) on subjective arousal. There was a main effect of time, such that arousal increased over time. Means and standard errors are shown. N=20 due to one missing datapoint.

### Figure S3b. ‘Want snack’ ratings during cue sessions

Effects of cue (active, neutral) on ‘want snack’ at the four timepoints (pre-visual cues, post-visual cues, pre-multisensory cues, post-multisensory cues) on days when snacks were available. On the active cue day, ‘want snack’ was higher after the multisensory cues compared to the previous two timepoints, but this was not the case on the neutral cue day. Means and standard errors are shown. N=21. *p<0.05.

### Figure S3c. ‘Want cannabis’ ratings

Effects of cue (active, neutral) on ‘want cannabis’ at the four timepoints (pre-visual cues, post-visual cues, pre-multisensory cues, post-multisensory cues) on days when cannabis was available. On the active cue day, ‘want cannabis’ increased from pre-visual cues to post-multisensory cues, but this did not happen on the neutral cue day. Data were analyzed non-parametrically, but are visualized with means and standard errors of the mean. N=20 due to one missing data point. *p<0.05.

### Figure S4. Mean indifference points from behavioral choice task

Calculated from the behavioral choice task, the US dollar value at which participants were as likely to choose 3 puffs of cannabis/3 snacks as the monetary option. Shown in black for the active cue conditions and in grey for the neutral cue conditions.

### Figure S5. One sample t-test of Cannabis > 0 across the whole brain (Options epoch)

The initial half of images from top left are right hemisphere and the second half of the images are the left hemisphere. This figure visualizes the results shown in table S6.

### Figure S6. Reward X cue interaction across the whole brain (Choice epoch)

The initial half of images from top left are right hemisphere and the second half of the images are the left hemisphere. This figure visualizes the results shown in table S7.

### Figure S7. Cannabis > Snack across the whole brain (Choice epoch)

The initial half of images from top left are right hemisphere and the second half of the images are the left hemisphere. This figure visualizes the results shown in table S8.

### Figure S8. Distribution of participants 

The CONSORT flow diagram showing the number of people who enrolled, completed the study, were included in behavioral analyses, and were included in MRI analyses.

# References

Beck, Steer, R.A., Brown, G.K., 1996. Manual for the Beck depression inventory-II. San Antonio, TX: Psychological Corporation.

Bedi, G., Cooper, Z.D., Haney, M., 2013. Subjective, cognitive and cardiovascular dose‐effect profile of nabilone and dronabinol in marijuana smokers. Addiction biology 18, 872–881.

Bedi, G., Hao, X., van Dam, N.T., Cooper, Z.D., Rubin, E., Vadhan, N.P., Marino, L., Haney, M., 2019. Social motivational processing and interpersonal function in aging cocaine smokers. Addiction Biology 24, 1044–1055.

Clithero, J.A., Rangel, A., 2014. Informatic parcellation of the network involved in the computation of subjective value. Soc Cogn Affect Neurosci 9, 1289–1302.

Copeland, J., Gilmour, S., Gates, P., Swift, W., 2005. The Cannabis Problems Questionnaire: factor structure, reliability, and validity. Drug Alcohol Depend 80, 313–319.

First, M.B., France, A., Pincus, H.A., 2004. DSM-IV-TR guidebook. American Psychiatric Publishing, Inc.

Haney, M., Bedi, G., Cooper, Z.D., Glass, A., Vosburg, S.K., Comer, S.D., Foltin, R.W., 2013. Predictors of marijuana relapse in the human laboratory: robust impact of tobacco cigarette smoking status. Biol Psychiatry 73, 242–248.

Hare, T.A., Camerer, C.F., Rangel, A., 2009. Self-control in decision-making involves modulation of the vmPFC valuation system. Science (1979) 324, 646–648.

Lang, P.J., Bradley, M.M., Cuthbert, B.N., 1997. International affective picture system (IAPS): Technical manual and affective ratings. NIMH Center for the Study of Emotion and Attention 1, 3.

Maier, S.U., Makwana, A.B., Hare, T.A., 2015. Acute stress impairs self-control in goal-directed choice by altering multiple functional connections within the brain’s decision circuits. Neuron 87, 621–631.

Ree, M.J., MacLeod, C., French, D., Locke, V., 2000. State-trait inventory for cognitive and somatic anxiety (STICSA). Australia: The University of Western Australia.

Resnick, H.S., Kilpatrick, D.G., Dansky, B.S., Saunders, B.E., Best, C.L., 1993. Prevalence of civilian trauma and posttraumatic stress disorder in a representative national sample of women. J Consult Clin Psychol 61, 984.

Selzer, M.L., Vinokur, A., van Rooijen, L., 1975. A self-administered short Michigan alcoholism screening test (SMAST). J Stud Alcohol 36, 117–126.

Stunkard, A.J., Messick, S., 1985. The three-factor eating questionnaire to measure dietary restraint, disinhibition and hunger. J Psychosom Res 29, 71–83.

Tom, S.M., Fox, C.R., Trepel, C., Poldrack, R.A., 2007. The neural basis of loss aversion in decision-making under risk. Science (1979) 315, 515–518.

Ventura, J., Liberman, R.P., Green, M.F., Shaner, A., Mintz, J., 1998. Training and quality assurance with the Structured Clinical Interview for DSM-IV (SCID-I/P). Psychiatry Res 79, 163–173.
